# Use of public and private oral health services by Brazilian adults from 2003 to 2023: trends and socioeconomic inequalities

**DOI:** 10.1590/1980-549720260006.supl.1

**Published:** 2026-07-24

**Authors:** Líria Sheila Chamane, Rosana Leal do Prado, Doralice Severo Cruz, Ana Luiza Guerra Francisco, Maria Luíza Viana Fonseca, Maria Luiza do Nascimento Silva, Loliza Luiz Figueiredo Houri Chalub, Raquel Conceição Ferreira

**Affiliations:** I Universidade Federal de Minas Gerais - Belo Horizonte (MG), Brazil.; II Ministério da Saúde, Secretaria de Vigilância em Saúde - São Paulo (SP), Brazil.

**Keywords:** Effective access to health services, Health inequities, Dental health services, Socioeconomic factors, Equity, Epidemiology

## Abstract

**Objective:**

To analyze socioeconomic inequalities in the use of oral health services among Brazilian adults aged 35-44 years between 2003 and 2023, according to level of education, income, and type of service used.

**Methods:**

This is a cross-sectional study on data from the National Survey of Oral Health (SB Brasil) 2003, 2010, and 2023. Weighted prevalences of the use of oral health services in the previous year were estimated, and absolute (Slope Index of Inequality - SII) and relative (Relative Index of Inequality - RII) measures of inequality were calculated according to level of education and income. Multiple logistic regression models were used to assess the association between socioeconomic indicators and service use.

**Results:**

Between 2003 and 2023, the proportion of adults who used oral health services in the previous year increased from 38.7 to 51.6%. The RII for level of education decreased from 2.16 to 1.39, and the SII, from 0.30 to 0.20, indicating a reduction in educational inequalities, statistically significant among public service users (RII=1.08; SII=0.07 in 2023). Income-related inequalities remained unchanged. According to adjusted models, there was a significant interaction between level of education and type of service, with a steeper gradient in the private sector and a more homogeneous pattern in the public sector.

**Conclusion:**

From 2003 to 2023, the use of oral health services increased, and educational inequalities declined among users of public services, reflecting progress in the consolidation of the National Oral Health Policy and in the expansion of public access. However, economic disparities persist, underscoring the need for redistributive policies that integrate equity and sustainability in the financing of the Brazilian Unified Health System.

## INTRODUCTION

The use of oral health services is a determinant of health, as it reflects the degree to which available resources translate into effective care^1^ ^,^ ^2^ ^,^ ^3^ ^,^ ^4^. In addition to meeting clinical needs, it indicates the ability of the system to provide timely, affordable, and continuous care. When regular, it favors prevention, early diagnosis, and treatment of oral diseases, being an essential component of healthcare systems. The type and opportunity of use express the accessibility, efficiency of the system, and the ability of individuals to seek and receive care^3^ ^,^ ^4^ ^,^ ^5^. From the perspective of equity, it is an indicator of access, quality, and distributive justice^5^.

Access to oral health care is conditioned by social, economic, and structural factors, which go beyond clinical needs^6^ ^,^ ^7^. Social gradients persist both in the occurrence of oral diseases^7^ ^,^ ^8^ ^,^ ^9^ and in the use of services^9^ ^,^ ^10^. People with higher levels of education and income have more preventive and rehabilitative consultations, while socially-disadvantaged groups, despite worse oral health conditions, tend to seek care only in pain or urgency situations^7^ ^,^ ^10^ ^,^ ^11^ ^,^ ^12^. Even in the context of the expansion of public coverage, such as the Brazilian’s, these inequalities persist^10^, which reflects the role of social stratification and material living conditions^10^ ^,^ ^13^. In this scenario, the health system acts as an intermediate determinant, with the potential to reduce inequities by promotion and prevention actions aimed at the most vulnerable groups^13^.

In Brazil, oral health care has historically shown strong participation of the private sector^14^ and limited public supply. The creation of the National Oral Health Policy (*Política Nacional de Saúde Bucal* - PNSB), in 2004, marked an inflection point in State action by proposing a model of comprehensive and continuous care within the Brazilian Unified Health System (SUS)^15^ ^,^ ^16^ ^,^ ^17^ ^,^ ^18^ ^,^ ^19^. Known as “Smiling Brazil” (*Brasil Sorridente*) and recently turned into Law 14.572/2023^18^, the policy promoted the expansion of Oral Health Teams in Primary Health Care, the creation of Dental Specialty Centers and Regional Dental Prosthesis Laboratories, in addition to strengthening surveillance actions such as community water fluoridation ^17^ ^,^ ^18^. These initiatives sought to ensure comprehensive care and reduce inequalities in access to services.

Despite advances in structuring public oral health care over the last two decades, regional and socioeconomic inequalities persist, which compromise equity in access to services^10^ ^,^ ^11^. Although underscoring these disparities, studies that analyze their temporal evolution based on representative population data still lack in the literature^11^ ^,^ ^20^. Comparative analyses of oral health surveys allow us to evaluate, from a longitudinal perspective, the possible contribution of public policies and the expansion of SUS coverage to reducing inequalities. In this study, we analyzed the magnitude and trends of socioeconomic inequalities in the use of oral health services among Brazilian adults aged 35-44 years, from 2003 to 2023, according to level of education, income, and type of service used, in addition to evaluating whether the type of service modified the association between the use of oral health services in the previous year according to education and income.

## METHODS

### Study design and data source

This is a cross-sectional study analyzing secondary data from the National Survey of Oral Health (SB Brasil), conducted in 2003, 2010, and 2023, in Brazil, reported according to the STROBE (Strengthening the Reporting of Observational Studies in Epidemiology) guideline. Participants in the age group of 35-44 years, recommended by the World Health Organization (WHO) for assessing oral health conditions of adults^21^, were included. The three surveys used complex probabilistic cluster samples. In 2003, the sampling domains corresponded to the five regions and their respective municipalities, using three- or four-stage sampling according to population size. The primary sampling units (PSUs) corresponded to 50 municipalities per region. In 2010, the sample plan included 32 domains (27 capitals and five inland regions), using two- or three-stage cluster sampling and selecting 30 PSUs per domain, ensuring a coefficient of variation <15% for >10% estimates. In 2023, 53 domains were considered (26 capitals and 27 Federation Units), adopting two-stage sampling (census tracts and households), with 400 adults investigated per Federation Unit, ensuring adequate sampling accuracy^22^ ^,^ ^23^ ^,^ ^24^ ^,^ ^25^.

### Data collection

Data were collected in the households through interviews and oral examinations performed by previously trained teams, according to criteria recommended by the WHO^21^ ^,^ ^23^. In 2003 and 2010, training and calibration of the teams comprised 32 hours of in-person theoretical-practical activities. In 2023, the theoretical content was made available on the Moodle^®^ platform, with the use of videos and photographic simulations for calibration^26^ ^,^ ^27^. Inter-rater agreement was evaluated by the weighted or simple Kappa coefficient, with acceptable minimum values of 0.65 (2003/2010) and 0.61 (2023)^23^ ^,^ ^26^. The three projects were approved by the National Commission of Ethics in Research (2003: 1.356; 2010: 15.498; 2023: 4.823.054). All participants signed the Informed Consent Form.

### Dependent variable

The outcome was the time since the last visit to the oral health service. In 2003 and 2010, participants answered whether they had used the oral health services once in their lives. Among those who did, the time since the last consultation was classified as <1 year, 1 to 2 years, and ≥3 years. In 2023, a single question included the option “had never used it” and the category “>2 to 3 years” was also included. For comparability purposes, a variable was created with the categories: had never used it; used it in the previous year; >1 to 3 years; and >3 years.

### Independent variables of interest and covariates

According to Andersen’s and Davidson’s model (1997)^3^ ^,^ ^11^ ^,^ ^20^ ^,^ ^28^, the variables included enabling factors (income, level of education, type of service used), predisposing factors (sex, age, and race/skin color), and need factors (toothache, presence of dental caries, tooth loss, and self-rated oral health). The independent variables of interest were family income, level of education, and type of service used. Family income was recorded in the Brazilian currency (reais, BRL) in the 2003 and 2023 surveys, and in predefined categories in 2010 (up to BRL 250; 251-500; 501-1,500; 1,501-2,500; 2,501-4,500; 4,501-9,500; >9,500). The values were converted into minimum wages (MW), considering BRL 200.00 (USD 69.56) in 2003; BRL 510.00 (USD 283.41) in 2010; and BRL 1,320.00 (USD 271.71) in 2023, and categorized into: up to 1; >1-3; >3-5; and >5 MW. The level of education variable was based on the complete years of formal study, without failing, and was categorized into 0, 1-4, 5-8, 9-11, and ≥12 years of formal study^29^ ^,^ ^30^ ^,^ ^31^. The type of service used, registered for those who had used oral health services once in their life, was classified as public or private service (private + health insurance/supplementary private insurance and others).

### Covariates

The race/skin color variable was self-reported according to the Brazilian Institute of Geography and Statistics: white, Black, mixed-race, Asian, and Indigenous. The Asian and Indigenous categories were grouped due to low frequency in the surveys.

Toothache was based on the report of occurrence in the last six months. The presence of dental caries was evaluated according to the WHO criteria^21^ and defined as the presence of ≥1 tooth with untreated dental caries or a restoration with caries. Tooth loss was based on the number of missing teeth (teeth lost due to caries or other reasons) according to the WHO^21^ and was categorized as <21 and ≥21 present teeth, considering the number of teeth needed to maintain basic oral functionality^32^ ^,^ ^33^. ‌Self-rated oral health was standardized among the surveys. In 2003, 2010, and 2023, participants evaluated their oral health or their degree of satisfaction through equivalent scales. All responses were grouped into three categories: positive (very good, good, very satisfied, satisfied), fair (fair, neither satisfied nor dissatisfied), and negative (very bad, bad, dissatisfied, very dissatisfied).

### Statistical analysis

All analyses were performed in Stata^®^, accounting for the complex sampling design and sample weights of each survey, including those of 2003, calculated by Queiroz et al.^22^. Adults’ percentages were estimated based on time since the last visit to oral health services, the survey year, and service type (public or private), with 95% confidence intervals (CIs). Estimates by type of service included only adults who used oral health services. The answers “I do not know/did not answer” were deemed losses.

Absolute differences between years (Δ2023-2010, Δ2023-2003, Δ2010-2003) were tested with the lincom command^34^. The annual percent change (APC) was calculated by exponential regression of the logarithm of the percentages as a function of time (ln(𝑌𝑡)=𝛼+𝛽𝑡, where APC=[exp(𝛽)−1]×100), with 95%CI obtained by the delta method (nlcom). The percentages of adults who used services in the previous year, by level of education and income, were represented on equiplot graphs. Inequalities were measured by the Slope Index of Inequality (SII) and the Relative Index of Inequality (RII)^35^. The ordinal categories of income and level of education were turned into ridit-score, representing the cumulative socioeconomic position. The indices were estimated by generalized linear regression, with the use of oral health services in the previous year as the dependent variable and the ridit-score as the independent variable, adjusted for all covariates. For the SII, the identity function and the binomial family were adopted; for the RII, the log function and the binomial family were adopted. The SII expresses the absolute difference between the extremes of the socioeconomic gradient (positive values indicate a higher prevalence among advantaged groups;negative values, among the disadvantaged). The RII represents the adjusted prevalence ratio (RII>1: greater use among the advantaged; RII=1: absence of inequality; RII<1: greater use among the disadvantaged). Both were calculated for the total sample and for subsamples (users of public or private services). Temporal trends were evaluated using interaction terms between ridit-score and survey year; significant coefficients indicated changes in inequalities over time^36^. The association between income, level of education, and use of services in the previous year was estimated using multiple logistic regression, with odds ratios and 95% CI reported. The models were adjusted for the covariates. A combined model gathered the three surveys, including interactions between years and socioeconomic variables, to test temporal stability. As they were not significant, the interactions between type of service and socioeconomic indicators were tested, gathering the three databases (Model 1: type of service × level of education; and Model 2: type of service × family income), adjusting for the survey year. Multicollinearity was assessed using the variance inflation factor (1.03-6.41; mean=2.29), with no indication of high collinearity. The non-significant result of the Hosmer-Lemeshow test indicated good fit, and the area under the ROC curve (AUC-ROC) demonstrated the overall discriminatory capacity of the models^37^.

The adjusted probabilities of service use in the previous year were estimated with the margins command and graphically displayed (marginsplot). The sensitivity analysis compared models with complete data for all variables and, after imputation, for income and level of education variables. Multiple imputation by chained equations assumed missing at random, generated 20 databases, and combined estimates and standard errors according to Rubin’s rules^38^ ^,^ ^39^.

## Data Availability Statement:

The dataset that supports the results of this study is not publicly available.

## RESULTS

A total of 13,430, 9,779, and 9,019 adults participated in the study in 2003, 2010, and 2023, respectively. Population estimates, stratified by sociodemographic characteristics and time since the last consultation, for Brazilian adults in 2003, 2010, and 2023, are presented in Table 1S (supplementary file). The absence of response for the variable “time since the last consultation” was 0.79%, 1.92%, and 3.65% for the respective years. Among those who had already used oral health services (2003: 12,946; 2010: 8,903; and 2023: 8,598), the percentage of complete answers for all variables were 94%, 93%, and 70%, respectively. Considering the three surveys (n=31,061), 86.6% presented complete data (Table 2S).

In all surveys, most adults reported having used oral health services in the previous year (Table 1), a percentage that increased from 38.7 to 51.6% between 2003 and 2023, while the percentage of use >3 years ago decreased from 35.3 to 15.1% (APC=-3.9%). The increase was significant among public service users, from 37.4 to 52.9% (APC=+1.74%), while private service users were not significant (APC=+0.99%) (Table 1).

**Table 1.** Percentage distribution of Brazilian adults according to time since the last visit to oral health services in the years 2003, 2010, and 2023, differences in percentages between the years (Δ), and annual percent change for the total sample and for those who had the last consultation in the public or private service. Brazil.

Time since the last visit to oral health services	Epidemiological surveys			Differences between estimates						Annual percent change	p-value
	2003 (n=13,324)%w (95%CI)	2010 (n=9,591)%w (95%CI)	2023 (n=8,690)%w (95%CI)	Δ2023-2010	p-value	Δ2023-2003	p-value	Δ2010-2003	p-value		
Had never used it	2.56 (1.95; 3.35)	7.28 (5.74; 9.19)	0.89 (0.44; 1.74)	-6.39 (-8.21; -4.57)	<0.001	-1.67 (-2.58; -0.75)	<0.001	4.72 (2.88; 6.57)	<0.001	-6.44% (-20.98; 8.10)	0.385
Previous year	38.69 (37.09; 40.31)	45.80 (43.85; 47.76)	51.62 (48.27; 54.96)	5.83 (1.95; 9.70)	0.003	12.93 (9.22; 16.65)	<0.001	7.11 (4.57; 9.64)	<0.001	+1.39 (0.63; 2.14)	<0.001
>1 to 3 years	23.44 (22.17; 24.76)	25.76 (23.70; 27.94)	32.35 (29.43; 35.42)	6.59 (2.92; 10.27)	<0.001	8.91 (5.65; 12.18)	<0.001	2.32 (-0.02; 4.81)	0.067	+1.64 (+1.44; 1.85)	<0.001
>3 years	35.31 (33.58; 37.09)	21.16 (19.50; 22.92)	15.13 (12.91; 17.65)	-6.03 (-8.95; -3.11)	<0.001	-20.18 (-23.13; -17.23)	<0.001	-14.15 (-16.60; -11.70)	<0.001	-3.95 (-6.22; -1.67)	0.001
Type of service used											
Public											
	2003 (n=6,405)	2010 (n=3,521)	2023 (n=4,130)								
Previous year	37.36 (35.85; 38.88)	42.63 (38.87; 46.38)	52.92 (48.12; 57.72)	10.29 (4.19; 16.39)	0.001	15.55 (10.52; 20.59)	<0.001	5.26 (1.21; 9.31)	0.001	+1.75 (+1.63; +1.85)	<0.001
>1 to 3 years	22.46 (20.74; 24.19)	29.96 (26.58; 33.34)	31.84 (27.87; 35.81)	1.19 (-3.33; 7.10)	0.479	9.38 (5.06; 13.70)	0.001	7.50 (3.70; 11.29)	<0.001	+1.60 (-0.26; 3.44)	0.091
>3 years	40.17 (38.23; 42.12)	27.41 (24.25; 30.28)	15.24 (11.79; 18.69)	-12.18 (-16.66; -7.69)	<0.001	-24.93 (-28.90; -20.97)	<0.001	-12.76 (-16.23; -9.29)	<0.001	-4.69 (-5.14; -4.24)	<0.001
Private											
	2003 (n=6,520)	2010 (n=5,358)	2023 (n=4,390)								
Previous year	41.34 (51.26; 56.01)	53.64 (51.26; 56.01)	52.16 (47.68; 56.64)	-1.47 (-6.54; 3.59)	0.568	10.82 (5.69; 15.96)	<0.001	12.30 (8.84; 15.76)	0.001	+0.99 (-0.99; 2.98)	0.327
>1 to 3 years	25.09 (22.99; 27.19)	26.45 (23.93; 28.96)	33.22 (29.31; 37.13)	6.77 (2.12; 11.43)	0.004	8.13 (3.70; 12.57)	<0.001	1.36 (-1.91; 4.63)	0.415	+1.46 (0.95; 1.97)	<0.001
>3 years	33.58 (30.98; 36.18)	19.92 (17.85; 21.98)	14.62 (11.38; 17.85)	-5.30 (-9.14; -1.45)	0.007	-18.95 (-23.11; -14.80)	<0.001	-13.66 (-16.98; -10.34)	0.001	-3.86 (-6.30; -1.41)	0.002

Estimates weighted by the effect of the sampling design and sampling weights. The answers “I do not know” and “did not answer” were deemed losses, and the percentages were calculated considering only the total of valid responses.

**Figure 1.**
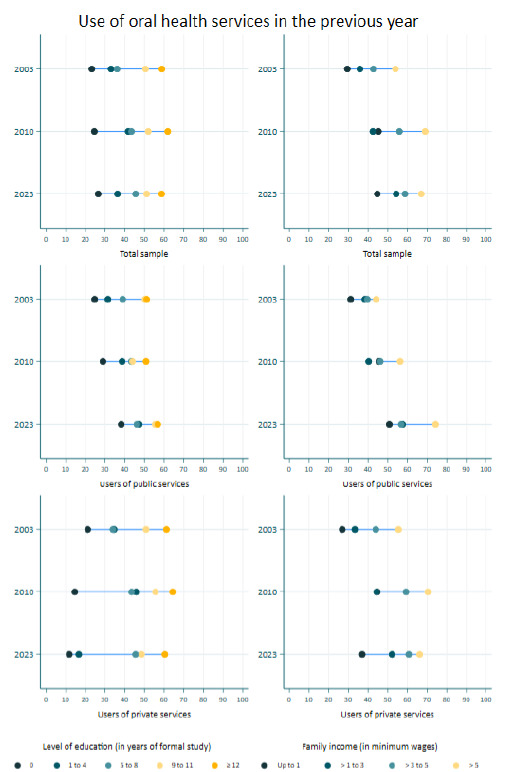
Equiplots of the prevalence of oral health services utilization in the previous year according to family income and level of education for the total sample and adults who had the last consultation in public or private services. Brazil.

According to the equiplot graphs, there was an approximation of levels of education over time, suggesting a reduction in inequalities in the use of services in the previous year (Figure 1, Table 3S). Among users of the public service, convergence was more evident, driven by increased use among the less educated. For the total sample, the RII decreased from 2.16 in 2003 to 1.39 in 2023. Among public service users, the RII decreased from 2.17 to 1.08; and the SII, from 0.27 to 0.07, indicating a reduction in relative and absolute disparities by level of education. Among private service users, there were no significant changes.

Estimates weighted by the effect of the sampling design and sampling weights.

As for income, considering the equiplot, there was stability in distances between socioeconomic strata. For users of private services, we observed higher rates of service use in the previous year among those with higher income, whose rates were distant from those with income of up to 1 MW. For public service users, this pattern was also noticed, but higher rates of service use in the previous year were observed in the group with up to 1 MW, compared to those who reported using the private sector. There was no significant reduction in relative or absolute inequalities by income (Table 2, Figure 1).

**Table 2.** Slope Index of Inequality and Relative Index of Inequality, with 95% confidence intervals, for the use of oral health services in the previous year, according to level of education and income among adults aged 35 to 44 years. Brazil (2003, 2010, and 2023).

Population analyzed	Epidemiological surveys			Coefficient of the ridit-score × survey interaction	p-value for the trend
	2003	2010	2023		
LEVEL OF EDUCATION					
	Relative Index of Inequality (RII) per level of education (95%CI)				
Total sample	2.16 (1.93; 2.42)	1.56 (1.32; 1.85)	1.39 (1.11; 1.73)	0.80 (0.69; 0.91)	0.001
Used public services	2.17 (1.92; 2.46)	1.27 (0.97;1.65)	1.08 (0.83; 1.40)	0.74 (0.64; 0.85)	<0.001
Used private services	2.19 (1.91; 2.51)	1.58 (1.25; 2.02)	1.73 (1.20; 2.48)	0.89 (0.70; 1.13)	0.339
	Slope Index of Inequality (SII) per level of education (95%CI)				
Total sample	0.30 (0.25; 0.34)	0.22 (0.14; 0.30)	0.20 (0.08; 0.31)	-0.06 (-0.13; 0.004)	0.067
Used public services	0.27 (0.22; 0.33)	0.11 (0.004; 0.23)	0.07 (-0.08; 0.22)	-0.08 (-0.16; -0.02)	0.019
Used private services	0.28 (0.22; 0.34)	0.25 (0.13; 0.36)	0.26 (0.08; 0.45)	-0.02 (-0.13; 0.08)	0.663
INCOME					
	Relative Index of Inequality (RII) per income (95%CI)				
Total sample	1.82 (1.54; 2.15)	1.63 (1.37; 1.93)	1.61 (1.31; 1.98)	0.91 (0.79; 1.04)	0.176
Used public services	1.34 (1.17; 1.61)	1.11 (0.85; 1.43)	1.24 (0.89; 1.72)	0.95 (0.79; 1.14)	0.566
Used private services	2.09 (1.60; 2.74)	1.72 (1.43; 2.07)	2.15 (1.53; 3.02)	0.97 (0.76; 1.23)	0.798
	Slope Index of Inequality (SII) per income (95%CI)				
Total sample	0.24 (0.18; 0.30)	0.25 (0.17; 0.33)	0.25 (0.14; 0.37)	-0.01 (-0.07; 0.06)	0.991
Used public services	0.12 (0.05; 0.18)	0.05 (-0.07; 0.16)	0.15 (-0.06; 0.36)	0.04 (-0.10; 0.11)	0.944
Used private services	0.30 (0.20; 0.39)	0.28 (0.19; 0.37)	0.38 (0.23; 0.55)	0.02 (-0.09; 0.12)	0.728

Estimates weighted by the sampling design and sampling weights. Models adjusted for sex, age, race/skin color, toothache in the previous month, dental caries, number of present teeth, and self-rated oral health. Answers “I do not know” and “did not answer” were deemed losses, and proportions were calculated considering only valid responses.

In the crude logistic regression, we identified a significant association between income, level of education, and use of services (Table 4S). There was no significant interaction between year and socioeconomic indicators, suggesting temporal stability of effects (Table 5S).

**Figure 2.**
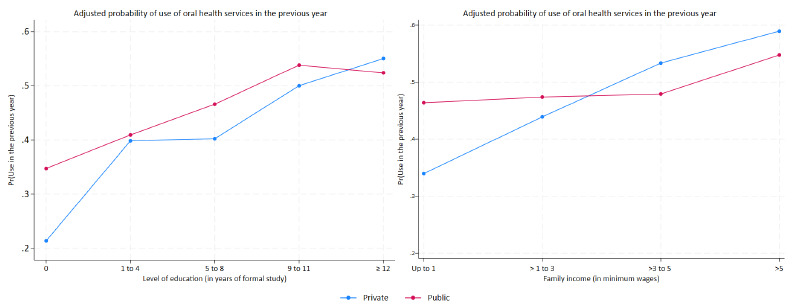
Adjusted predicted probabilities of use of oral health services in the previous year according to level of education and income, considering the type of service used.

In the adjusted model, we observed significant interactions between the type of service and both indicators, evidencing that the effect of income and education varied according to the type of service used. In Model 1 (level of education × type of service), the probability of use in the previous year increased with education, both among users of public and private services, with the steepest gradient among users of private services, ranging from 21.35 (0 years of formal study) to 55.06% (≥12 years). Among users of public services, it ranged from 34.75 to 52.41%, with a more horizontal line, indicating low inequality. In Model 2 (income × type of service), the probability of use in the previous year among public service users ranged from 46.39 (≤ MW) to 54.77% (>5 MW), while the private service ranged from 33.97 to 58.95%. Even with low income, users of the public service presented a higher probability of use in the previous year compared to those of similar income who used private services (Table 3, Figure 2).

**Table 3.** Association between level of education or income and the use of oral health services in the previous year among adults aged 35 to 44 years. Brazil (2003, 2010, and 2023).

Variables	Use of oral health services in the previous year	
	Model 1 (inclusion of the interaction between use of public service × level of education)	Model 2 (inclusion of the interaction between use of public service × family income)
	OR (95%CI)	OR (95%CI)
*Survey year*		
2003	1	1
2010	1.46 (1.28; 1.65)	1.46 (1.29; 1.65)
2023	1.68 (1.30; 2.17)	1.68 (1.30; 2.17)
*Age*	0.98 (0.95; 1.00)	0.98 (0.96; 1.00)
*Sex*		
Men	1	1
Women	1.39 (1.20; 1.61)	1.41 (1.22; 1.63)
*Race/skin color*		
White	1	1
Black	0.99 (0.83; 1.20)	0.99 (0.82; 1.19)
Asian + Indigenous	0.99 (0.75; 1.32)	0.99 (0.74; 1.32)
Mixed-race	0.95 (0.82; 1.10)	0.94 (0.82;1.09)
*Family income (minimum wages)*		
Up to 1	1	1
>1 to 3	1.21 (1.01; 1.47)	1.58 (1.04; 2.38)
>3 to 5	1.64 (1.34; 2.00)	2.39 (1.65; 3.44)
>5	2.20 (1.71; 2.83)	3.06 (2.06; 4.53)
*Level of education (years of formal study)*		
0	1	1
1 to 4	2.58 (1.39; 4.78)	1.66 (1.23; 2.24)
5 to 8	2.62 (1.49; 4.62)	1.90 (1.43; 2.52)
9 to 11	4.01 (2.28; 7.05)	2.74 (1.99; 3.79)
≥12	4.97 (2.67; 9.29)	3.10 (2.21; 4.35)
*Toothache in the last 6 months*		
No	1	1
Yes	2.28 (1.96; 2.64)	2.26 (1.95; 2.62)
*Presence of dental caries*		
Absent	1	1
≥1 carious tooth	0.84 (0.74; 0.96)	0.84 (0.74; 0.96)
*Number of present teeth*		
<21 teeth	1	1
≥21 teeth	0.76 (0.64; 0.92)	0.76 (0.64; 0.92)
*Self-rated oral health*		
Positive	1	1
Fair	0.81 (0.69; 0.95)	0.80 (0.69; 0.94)
Negative	0.52 (0.43; 0.63)	0.52 (0.42; 0.63)
*Type of service used*		
Private	1	1
Public	2.04 (1.07; 3.90)	1.76 (1.09; 2.84)
*Interactions between use of public service and level of education (years)*		
0	1	
1 to 4	0.52 (0.26; 1.04)	-
5 to 8	0.65 (0.33; 1.26)	-
9 to 11	0.58 (0.32; 1.05)	-
≥12	0.44 (0.21; 0.90)	-
*Interactions between use of public service and income (minimum wages)*		
Up to 1		
>1 to 3	-	0.66 (0.39; 1.13)
>3 to 5	-	0.45 (0.26; 0.77)
>5	-	0.47 (0.23; 0.95)
*n*	28,045	26,902

CI: Confidence interval; OR*:* Odds ratio.

Estimates weighted by the sampling design and sampling weights. Estimates obtained considering the sample of adults who used oral health services once in their lives. Bold values indicate statistically significant measures.

Predicted probabilities (Pr) from the adjusted logistic regression model. Model 1: (inclusion of the interaction between use of public service × level of education), adjusted for the variables survey year, sex, age, income, skin color, toothache, self-rated oral health, and number of present teeth. Model 2 (inclusion of the interaction between use of public service × family income), adjusted for the variables survey year, sex, age, level of education, skin color, toothache, self-rated oral health, and number of present teeth. Estimates weighted by the sampling design and sampling weights. Estimates obtained considering the sample of adults who used oral health services once in their lives.

The performance of the models was satisfactory, with good overall fit (Hosmer-Lemeshow test Model 1: F[9.1782]=0.67, p=0.7399; Model 2: F[9.1782]=1.17, p=0.3099). AUC ROC was 0.6661 (0.65981; 0.67247) for Model 1 and 0.6580 (0.65155; 0.66450) for Model 2, demonstrating moderate discriminatory capacity. The coefficients of the models with imputed data were similar to those obtained with the complete case sample for all variables (Table 6S).

## DISCUSSION

Although social gradients persist in access to dental care, in this study, we verified an increase in the use of oral health services in the previous year in Brazil between 2003 and 2023 and a reduction in inequalities in this use, according to level of education. As per the analysis stratified by type of service, a reduction of these inequalities was identified among users of public services. In the private sector, the educational gradient remained steep, reflecting patterns of social stratification. These findings suggest a positive effect of the consolidation of PNSB within the SUS and its contribution to the expansion of access, especially through the expansion of Oral Health Teams in Primary Health Care and the structuring of the Oral Health Network^16^ ^,^ ^17^ ^,^ ^40^.

Conversely, according to the results, there was lower use of services in the previous year among individuals with higher clinical demands, such as the presence of dental caries and negative self-rated oral health, suggesting delayed access, accumulation of needs, and persistence of barriers among more vulnerable groups. This pattern is corroborated by the greater use of services among people who reported a toothache. Most participants sought care for a toothache, tooth extraction, or treatment. However, among users of the public service in 2023, about 33% reported routine consultation as the main reason, an expressive advance in relation to the 19% observed in 2010 (results not shown). These findings highlight progress in the incorporation of preventive actions in the SUS, although the curative pattern remains predominant.

In this context, the expansion of coverage and the planning of public policies should consider the Inverse Care Law, ensuring that access and quality of care do not favor the most advantaged strata, but prioritize groups with greater needs^41^ ^,^ ^42^. The SUS plays a key role in standing against the market logic, which tends to reinforce inequalities and perpetuate the effects of this law. The increase in the search for services aimed at maintaining oral health may also reflect cultural changes in health behavior, as individuals who have periodic consultations tend to adopt more consistent self-care practices - such as reducing risk factors and increasing oral health capital throughout life^7^ ^,^ ^8^. The increasing trend of use of oral health services in the previous year and the search for timely care can contribute to modifying the epidemiological profile of the population, favoring more conservative care practices aimed at health promotion and prevention of diseases. Furthermore, this process can reduce the need for invasive procedures and strengthen the transition to a more comprehensive healthcare model^30^.

We verified consistent differences in the use of oral health services in the previous year, according to level of education and income, with an increase in prevalence between 2003 and 2023. The decrease of inequalities in education, especially among public service users, indicates the redistributive role of SUS and the advancement of universal oral health policies. This finding suggests partial overcoming of educational barriers, driven by the expansion of Primary Health Care and the incorporation of specialized services^20^ ^,^ ^30^. This process was strengthened by the recomposition of the federal financing of PNSB in 2023, via Ordinance GM/MS 1.924/2023^43^, which readjusted incentives to Oral Health Teams, Regional Dental Prosthesis Laboratories, and Dental Specialty Centers, contributing to reverse the historical underfunding, increase the response capacity of the SUS, especially in smaller municipalities and more vulnerable regions, and sustain equity and problem-solving capacity gains^44^.

These findings corroborate evidence that, by reorganizing the oral healthcare model, universal systems can reduce inequities linked to education, even if economic disparities persist^28^. Income inequalities remained practically unchanged in the two decades analyzed. Despite the expansion of public supply, family income continued to influence the use of services, especially private ones, confirming the role of the enabling factors, according to which economic capacity is an essential determinant of access. Direct and indirect financial barriers, such as transport costs, loss of working days, and material expenses, still limit the use of services by lower-income groups, restricting the potential of public policies to promote economic equity^3^.

The effects of income on the reduction of inequities are complex and reflect the limitations of the mechanisms of redistribution in the Brazilian context. Although programs - such as *Bolsa Família* (cash transfer program for low-income families from the Brazilian government) - have contributed to reducing poverty and improving health and education indicators^45^, the concentration of income in the country remains among the largest in the world. This persistence is partly explained by the regressive tax structure, which disproportionately burdens the poorest, and the increase in informal employment, which reduces tax collection and weakens social protection^46^ ^,^ ^47^. Within this context, income remains a structural determinant of health inequities^13^, unevenly influencing access to oral health services and the effective use of care. Tax justice policies and the expansion of social investment - health, education, and social protection - play a key role in mitigating inequalities^46^.

Limitations include possible memory bias resulting from self-reported information and the absence of contextual variables in the model. Cluster sampling can underestimate standard errors in small subgroups, as well as allowing for under-representation of individuals without access to services. The analyses are based on three moments, which reduces the power to detect gradual changes in inequalities. The tests of interaction between the ridit-score and year evaluate statistical variations over time, but do not allow causal inferences or the identification of underlying mechanisms such as the specific impact of public policies or other contextual changes. The calculation of APC presupposes a constant exponential pattern and may not capture discontinuities resulting from economic or institutional changes. Inequalities were measured by the RII and SII, given their temporal comparability when considering the relative position of socioeconomic categories^35^ ^,^ ^36^ ^,^ ^48^. Although demographic changes may affect population composition, these indices are suitable for trend analysis by incorporating the entire socioeconomic distribution. Family income was used because it was compatible between the surveys. However, it should be noted that the equivalent per capita income is recommended for economic stratification and poverty analysis, with impacts on income-oral health associations^49^. Nonetheless, its application was made unfeasible by the different forms of income collection (2010: categorical; 2003/2023: continuous), compromising temporal comparability, the main objective of the analysis.

Between 2003 and 2023, there was an increase in the use of oral health services in the previous year and a reduction in inequalities according to level of education, suggesting advances in the consolidation of PNSB and the expansion of public access. Nevertheless, economic inequalities persist, highlighting the need for redistributive policies that integrate equity and sustainability into SUS financing.

## Supplementary Material

Supplementary Material
