# Supplementary material for: Use of public and private oral health services by Brazilian adults from 2003 to 2023: trends and socioeconomic inequalities
Source: Rev Bras Epidemiol. 2026 Jul 24;29(Suppl 1):e260006supl1. doi: 10.1590/1980-549720260006.supl.1 (PMC13440361; doi:10.1590/1980-549720260006.supl.1)
Supplement: Supplementary file 1 [file 1980-5497-rbepid-29-suppl1-e260006supl1-sppl.pdf]

Tabelas suplementares

Utilização de serviços de saúde bucal públicos e privados no Brasil de 2003 a 2023: tendências e desigualdades socioeconômicas

Tabela 1S: Distribuição dos adultos segundo variáveis sociodemográficas e de saúde bucal nos anos de 2003, 2010 e 2023 para amostra total e entre aqueles que utilizaram serviço de saúde bucal no último ano. Brasil.

	2003			2010			2023		
		**Amostra total**	**Utilização de serviços de saúde bucal no último ano**		**Amostra total**	**Utilização de serviços de saúde bucal no último ano**		**Amostra total**	**Utilização de serviços de saúde bucal no último ano**
	**n**	**%w (95% IC)**	**%w (95% IC)**	**n**	**%w (95% IC)**	**%w (95% IC)**	**n**	**%w (95% IC)**	**%w (95% IC)**
Sexo									
Masculino	4353	48,54 (47,18; 49,91)	34,32 (32,23; 36,47)	3374	36,87 (34,40; 39,41)	41,87 (38,85; 44,96)	2876	33,71 (31,19; 36,32)	46,58 (41,33; 51,90)
Feminino	9077	51,46 (50,09; 52,82)	42,81 (40,98; 44,67)	6405	63,13 (60,59; 65,60)	48,07 (45,67; 50,47)	6143	66,29 (63,68; 68,81)	54,17 (50,24; 58,05)
Escolaridade (em anos de estudo)									
0	1001	6,32 (5,80; 6,88)	21,85 (19,61; 24,26)	146	1,06 (0,82; 1,35)	20,27 (14,60; 27,43)	135	1,77 (1,11; 2,80)	23,98 (10,94; 44,76)
1 a 4	4602	29,76 (27,93; 31,66)	31,61 (29,22; 34,11)	1507	19,77 (17,65; 22,09)	37,09 (32,94; 41,44)	346	4,82 (3,39; 6,81)	35,32 (23,85; 48,77)
5 a 8	4033	35,08 (32,71; 37,54)	35,42 (32,51; 38,45)	2718	29,01 (27,17; 30,92)	39,88 (35,94; 43,95)	1153	12,96 (10,98; 15,23)	45,15 (35,72; 54,95)
9 a 11	2318	20,69 (18,90; 22,60)	49,99 (47,65; 52,32)	3025	28,99 (26,90; 31,18)	49,25 (45,18; 53,33)	3154	38,07 (33,54; 42,82	50,80 (46,13; 55,45)
≥ 12	1192	8,15 (6,76; 9,80)	58,28 (52,22; 64,09)	2297	21,17 (18,25; 24,42)	59,01 (54,36; 63,50)	4130	42,39 (37,48; 47,45)	58,36 (54,27; 62,34)
Renda em salários mínimos									
Até 1	2692	13,65 (12,47; 14,92)	27,83 (24,86; 31,00)	1420	13,06 (11,51; 14,79)	37,22 (32,61; 42,09)	1737	26,19 (21,33; 31,72)	44,12 (37,79; 50,65)
> 1 a 3	6488	46,29 (43,95; 48,66)	34,76 (32,32; 37,28)	4783	53,13 (49,68; 56,56)	39,68 (36,84; 42,60)	3055	45,55 (40,30; 50,90)	54,11 (47,76; 60,33)
> 3 a 5	2296	22,47 (20,34; 24,75)	42,05 (39,71; 44,44)	1846	20,61 (18,53; 22,85)	53,19 (48,37; 57,96)	1128	19,36 (16,26; 22,89)	58,62 (52,21; 64,76)
> 5	1810	17,59 (15,60; 19,77)	53.70 (48,75; 58,57)	1486	13,20 (11,08; 15,65)	66,27 (60,41; 71,66)	725	8,89 (6,44; 12,16)	66,93 (56,71; 75,77)
Raça/cor da pele									
Branca	5893	45,37 (42,89; 47,87)	43,63 (40,42; 46,89)	4137	50,15 (46,89; 53,40)	50,05 (47,47; 52,64)	2848	42,24 (38,30; 46,28)	52,17 (47,30; 57,00)
Preta	1231	9,42 (8,24; 10,75)	36,41 (32,54; 40,46)	1020	10,85 (9,54; 12,32)	39,63 (32,77; 46,92)	1184	13,49 (11,28; 16,06)	50,41 (43,20; 57,61)
Amarela + indígena	587	3,76 (3,17; 4,45)	38,58 (33,11; 44,35)	236	1,67 (1,30; 2,15)	26,25 (19,37; 34,53)	164	1,64 (1,10; 2,44)	51,58 (33,31; 69,44)
Parda	5686	41,45 (39,43; 43,50)	33,81 (31,76; 35,93)	4386	37,33 (34,51; 40,23)	42,75 (39,24; 46,34)	4745	42,64 (38,65; 46,72)	51,54 (46,85; 56,20)
Dor dentária nos últimos 6 meses									
Não	8719	62,19 (60,45; 63,89)	35,26 (33,48; 37,08)	7324	72,19 (70,22; 74,09)	43,58 (41,07; 46,12)	6697	76,35 (73,24; 79,20)	46,83 (43,32; 50,37)
Sim	4660	37,81 (36,11; 39,55)	44,35 (42,07; 46,67)	2366	27,81 (25,91; 29,78)	51,94 (48,12; 55,74)	2237	23,65 (20,80; 26,76)	67,72 (62,45; 72,58)
Presença de cárie dentária									
Ausente	5175	36,69 (34,47; 38,96)	43,56 (40,85; 46,31)	3819	45,62 (43,59; 47,66)	52,22 (49,07; 55,35)	4014	47,26 (43,79; 50,75)	54,29 (49,84; 58,68)

	2003			2010			2023		
		Amostra total	Utilização de serviços de saúde bucal no último ano		Amostra total	Utilização de serviços de saúde bucal no último ano		Amostra total	Utilização de serviços de saúde bucal no último ano
	n	%w (95% IC)	%w (95% IC)	n	%w (95% IC)	%w (95% IC)	n	%w (95% IC)	%w (95% IC)
≥ 1 dente cariado	8255	63,31 (61,04; 65,53)	35,87 (34,46; 37,30)	5745	54,38 (52,34; 56,41)	40,52 (38,17; 42,92)	4973	52,74 (49,25; 56,21)	49,19 (44,85; 53,55)
Número de dentes presentes									
< 21 dentes	8719	69,63 (68,13; 71,09)	42,37 (40,14; 44,63)	6189	63,99 (61,06; 66,82)	47,81 (45,84; 49,79)	2454	29,85 (26,85; 33,04)	52,13 (45,69; 58,49)
≥ 21 dentes	4660	30,37 (28,91; 31,87)	30,24 (27,41; 33,22)	3590	36,01 (33,18; 38,94)	42,11 (38,73; 45,56)	6565	70,15 (66,96; 73,15)	51,40 (47,98; 54,80)
Autopercepção da saúde bucal									
Positiva	5129	36,06 (34,37; 37,78)	43,77 (41,90; 45,66)	3905	40,87 (38,59; 43,20)	51,02 (46,45; 55,58)	4294	49,05 (45,92; 52,20)	57,83 (52,86; 62,65)
Regular	4963	39,67 (38,16; 41,19)	40,38 (37,53; 43,30)	1779	20,88 (19,02; 22,89)	46,95 (42,09; 51,88)	3163	35,67 (32,88; 38,56)	48,97 (44,61; 53,35)
Negativa	2953	24,28 (23,08; 25,51)	30,85 (27,31; 34,64)	4009	38,24 (35,58; 40,98)	39,71 (37,10; 42,38)	1416	15,28 (13,21; 17,59)	39,72 (31,85; 48,17)

Estimativas ponderadas pelo delineamento amostral e pelos pesos de amostragem.

Tabela 2S: Distribuição de dados perdidos para as variáveis analisadas nos anos de 2003, 2010 e 2023. Brasil

	Inquéritos epidemiológicos		
	2003	2010	2023
**Amostra total de adultos em cada inquérito**	**13.430**	**9.779**	**9.019**
Adultos que nunca utilizaram serviços de saúde bucal	378	688	101
**Número de adultos considerados na análise de dados perdidos (n=31061)**	**13.052**	**9.091**	**8.918**
Número e percentual de registros completos para as variáveis analisadas			
Sexo	13.052 (100)	9.091 (100)	8.918 (100)
Idade	13.052 (100)	9.091 (100)	8.918 (100)
Escolaridade	12.771 (97,85)	9.018 (99,20)	8.820 (98,90)
Renda	12.911 (98,92)	8.861 (97,47)	6.584 (73,83)
Raça/cor da pele	13.021 (99,76)	9.091 (100)	8.840 (99,13)
Dor de dente	13.002 (99,62)	9.010 (99,10)	8.833 (99,05)
Cárie dentária	13.052 (100)	8.892 (97,81)	8.887 (98,37)
Autopercepção da saúde bucal	12.716 (97,43)	9.017 (99,19)	8.773 (98,37)
Tempo desde a última consulta	12.946 (99,19)	8.903 (97,93)	8.589 (96,31)
Tipo de serviços utilizados	12.977 (99,43)	8.978 (98,76)	8.522 (95,56)

Tabela 3S: Prevalência de utilização de serviços de saúde bucal no último ano entre adultos de 35 a 44 anos de idade, segundo escolaridade e renda familiar, para a amostra total e para usuários de serviços públicos e privados, nos anos de 2003, 2010 e 2023

	2003			2010			2023		
	Amostra total	**Público**	**Privado**	Amostra total	**Público**	**Privado**	**Amostra total**	**Público**	**Privado**
Escolaridade (em anos de estudo)									
0	23,27 (20,82; 25,72)	24,69 (22,02; 27,36)	21,15 (15,81; 24,49)	24,54 (18,44; 30,64)	28,91 (17,08; 40,73)	14,63 (2,55; 26,71)	26,46 (6,83; 46,10)	38,21 (15,46; 60,95)	11,81 (1,20; 29,22)
1 a 4	33,02 (30,48; 35,56)	31,44 (28,89; 33,99)	34,56 (31,96; 37,17)	41,73 (37,59; 45,88)	38,65 (34,90; 42,40)	45,77 (38,42; 53,13)	36,27 (23,18; 49,36)	47,33 (33,03; 61,63)	16,61 (1,13; 32,10)
5 a 8	36,07 (33,03; 39,10)	38,93 (35,95; 41,92)	34,00 (30,19; 37,81)	43,39 (39,09; 47,69)	43,36 (38,81; 47,91)	43,47 (37,15; 49,79)	45,47 (35,66; 55,27)	46,18 (37,02; 55,35)	45,46 (26,23; 64,69)
9 a 11	50,55 (48,18; 52,91)	50,31 (46,36; 54,26)	50,84 (47,69; 54,00)	51,91 (47,62; 56,21)	43,86 (35,09; 52,63)	55,63 (51,48; 59,77)	51,12 (46,44; 55,80)	55,47 (48,47; 62,48)	48,52 (42,70; 54,34)
≥ 12	58,88 (52,94; 64,81)	51,09 (45,79; 56,40)	61,17 (54,62; 67,72)	61,85 (56,49; 67,21)	50,82 (43,20; 58,45)	64,28 (58,42; 70,13)	58,76 (54,68; 62,83)	56,75 (50,74; 62,76)	60,39 (55,35; 64,44)
Renda em salários mínimos									
0 a 1	29,44 (26,21; 32,68)	31,14 (27,75; 34,52)	26,87 (21,82; 31,83)	45,25 (40,37; 50,13)	45,51 (41,09; 49,92)	44,66 (36,81; 52,51)	44,67 (38,06; 51,29)	50,78 (42,52; 59,05)	37,03 (23,91; 50,14)
> 1 a 3	35,85 (33,30; 38,40)	38,39 (36,70; 40,08)	33,25 (28,24; 38,26)	42,42 (39,32; 45,52)	40,29 (34,42; 46,16)	44,35 (40,75; 47,94)	54,27 (47,95; 60,60)	57,51 (50,0; 65,03)	52,18 (43,63; 60,74)
> 3 a 5	42,65 (40,33; 44,97)	39,57 (35,21; 49,93)	43,92 (40,18; 47,67)	55,95 (50,38; 61,52)	46,18 (38,34; 54,02)	59,25 (51,81; 66,69)	58,75 (52,45; 65,05)	56,87 (44,97; 68,77)	60,67 (52,93; 68,39)
> 5	53,89 (48,96; 58,82)	44,16 (38,12; 50,20)	55,49 (50,21; 60,76)	69,00 (63,66; 74,34)	56,33 (39,07; 73,58)	70,35 (65,25; 75,46)	67,04 (57,39; 76,68)	74,32 (46,23; 92,42	66,22 (56,81; 75,63)

Estimativas obtidas considerando a amostra dos adultos que utilizaram os serviços de saúde bucal alguma vez na vida. Estimativas ponderadas pelo delineamento amostral e pelos pesos de amostragem.

Tabela 4S: Associação de escolaridade e renda familiar com utilização de serviços de saúde bucal no último ano, entre adultos de 35 a 44 anos. Brasil (2003, 2010 e 2023)

Variáveis	2003<br>Odds Ratio bruta (IC 95%)	2010<br>Odds Ratio bruta (IC 95%)	2023<br>Odds Ratio bruta (IC 95%)
Escolaridade (em anos de estudo)			
0	1	1	1
1 a 4	**1,63 (1,35;1,96)**	**2,20 (1,52;3,20)**	1,58 (0,48;5,22)
5 a 8	**1,86 (1,54;2,24)**	**2,36 (1,62;3,43)**	2,32 (0,89;6,03)
9 a 11	**3,37 (2,86;3,98)**	**3,32 (2,29;4,81)**	**2,91 (1,03;8,21)**
>=12 anos de estudo	**4,72 (3,60;6,19)**	**4,99 (3,35;7,42)**	**3,96 (1,39;11,26)**
Renda familiar em salários mínimos			
0 a 1 sm	1	1	1
1 a 3 sm	**1,34 (1,10;1,63)**	0,89 (0,70;1,14)	**1,47 (1,02;2,12)**
3 a 5 sm	**1,78 (1,49;2,14)**	**1,54 (1,12;2,11)**	**1,76 (1,22;2,56)**
5 sm e +	**2,80 (2,21;3,55)**	**2,69 (1,97;3,69)**	**2,52 (1,51;4,19)**

Estimativas ponderadas pelo delineamento amostral e pelos pesos de amostragem. Estimativas obtidas considerando a amostra dos adultos que utilizaram os serviços de saúde bucal alguma vez na vida. Valores em negrito indicam medidas estatisticamente significativas.

Tabela 5S: Modelos ajustados de regressão logística para a utilização de serviços de saúde bucal no último ano testando interação entre renda (modelo 1) ou escolaridade (modelo 2) e ano do inquérito. Brasil.

Variáveis	Utilização de serviço de saúde bucal no último ano	
	Modelo 1 (Interação ano##renda familiar)	Modelo 2 (Interação ano##escolaridade)
	Odds Ratio ajustada	*Odds Ratio* ajustada
Ano do inquérito		
2003	1	1
2010	1,96 (1,48; 2,62)	1,71 (1,14; 2,55)
2023	1,60 (1,13; 2,28)	1,64 (0,51; 5,31)
Idade	0,98 (0,96; 1,00)	0,98 (0,95; 1,00)
Sexo		
Masculino	1	1
Feminino	1,41 (1,22; 1,63)	1,41 (1,22;1,63)
Escolaridade (em anos de estudo)		
0	1	1
1 a 4	**1,69 (1,21; 2,34)**	**1,61 (1,32;1,95)**
5 a 8	**1,91 (1,41; 2,58)**	**1,64 (1,31;2,04)**
9 a 11	**2,78 (1,99;3,89)**	**2,99 (2,46; 3,64)**
≥ 12	**3,17 (2,22; 4,53)**	**3,79 (2,84; 5,05)**
Renda familiar (em salários mínimos)		
Até 1	1	1
> 1 a 3	1,19 (0,98;1,44)	1,18 (0,95;1,48)
> 3 a 5	**1,35 (1,07; 1,71)**	**1,51 (1,23; 1,86)**
> 5	**1,71 (1,42; 2,07)**	**1,91 (1,55; 2,34)**
Raça/cor da pele		
Branca	1	1
Preta	1,00 (0,84; 1,21)	0,99 (0,84; 1,19)
Amarela + indígena	1,00 (0,76; 1,32)	0,99 (0,75;1,32)
Preta	0,95 (0,82;1,11)	0,95 (0,81; 1,10)
Dor dentaria nos últimos 6 meses		
Não	1	1
Sim	2,28 (1,97; 2,63)	2,29 (1,96; 2,66)
Presença de cárie dentária		
Não	1	1
≥ 1 dente cariado	0,85 (0,75; 0,96)	0,85 (0,75; 0,96)
Número de dentes presentes		
< 21 dentes	1	1
≥ 21 dentes	0,78 (0,64; 0,94)	0,78 (0,66; 0,94)

Variáveis	Utilização de serviço de saúde bucal no último ano	
	Modelo 1 (Interação ano##renda familiar)	Modelo 2 (Interação ano##escolaridade)
	Odds Ratio ajustada	*Odds Ratio* ajustada
Autopercepção da saúde bucal		
Negativa	1	1
Regular	0,80 (0,69; 0,94)	0,80 (0,68; 0,93)
Positiva	0,51 (0,42; 0,62)	0,50 (0,41; 0,61)
Ano do inquérito # Renda familiar		
2010#até 1	1	
2010#> 1 a 3	0,71 (0,49;1,01)	-
2010#> 3 a 5	0,87 (0,57;1,34)	-
2010#> 5	1,11 (0,75;1,64)	-
2023#até 1	1	
2023#> 1 a 3	1,09 (0,74; 1,62)	-
2023#> 3 a 5	1,21 (0,79; 1,84)	-
2023#> 5	1,24 (0,75; 2,04)	-
Ano do inquérito # Escolaridade		
2010#0		1
2010#1 a 4	-	1,08 (0,68; 1,72)
2010#5 a 8	-	1,16 (0,73;1,84)
2010#9 a 11	-	0,79 (0,51; 1,22)
2010#≥ 12	-	0,81 (0,48; 1,35)
2023#0		1
2023#1 a 4	-	1,11 (0,27; 4,61)
2023#5 a 8	-	1,53 (0,49; 4,80)
2023#9 a 11	-	0,98 (0,30; 3,25)
2023#≥ 12	-	0,89 (0,27; 2,97)
N	26.978	2.6978

Estimativas ponderadas pelo delineamento amostral e pelos pesos de amostragem. Estimativas obtidas considerando a amostra dos adultos que utilizaram os serviços de saúde bucal alguma vez na vida.

Tabela 6S: Associação entre escolaridade ou renda e a utilização de serviços de saúde bucal no último ano entre adultos de 35 a 44 anos. Brasil (2003, 2013 e 2023)

Variáveis	Modelo 1 (Interação renda familiar## uso de serviço público	Modelo 2 (Interação escolaridade## uso de serviço público
	Odds Ratio ajustada (IC 95%)	*Odds Ratio* ajustada (IC 95%)
Ano do levantamento		
2003	1	1
2010	**1,44 (1,27; 1,62)**	**1,43 (1,27; 1,62)**
2023	**1,48 (1,18; 1,86)**	**1,47 (1,17; 1,85)**
Idade	*0,98 (0,96;1,00)*	*0,98 (0,96;1,01)*
Sexo		
Masculino	1	1
Feminino	**1,36 (1,19;1,56)**	**1,35 (1,17; 1,55)**
Raça/Cor da pele		
Branca	1	1
Preta	0,99 (0,82; 1,21)	1,00 (0,82; 1,42)
Amarela + indígena	0,92 (0,69; 1,24)	0,93 (0,69; 1,24)
Parda	0,93 (0,81; 1,08)	0,94 (0,81; 1,09)
Renda familiar (em salários mínimos)		
Até 1	1	1
>1 a 3	**1,50 (1,01; 2,22)**	1,18 (0,98; 1,42)
> 3 a 5	**2,15 (1,47; 3,12)**	**1,53 (1,24; 1,90)**
> 5	**2,69 (1,81; 3,98)**	**1,97 (1,50; 2,59)**
Escolaridade (em anos de estudo)		
0	1	1
1 a 4	**1,58 (1,15; 2,17)**	**2,43 (1,30; 4,56)**
5 a 8	**1,85 (1,37; 2,52)**	**2,56 (1,42; 4,60)**
9 a 11	**2,72 (1,94; 3,81)**	**3,99 (2,21; 7,19)**
≥ 12	**3,21 (2,26; 4,54)**	**5,13 (2,74; 9,61)**
Dor dentária no último mês		
Não	1	1
Sim	**2,39 (2,08; 2,74)**	**2,40 (2,09; 2,76)**
Presença de cárie dentária		
Ausente	1	1
≥ 1 dente cariado	**0,85 (0,75; 0,96)**	**0,85 (0,75; 0,96)**
Número de dentes presentes		
< 21 dentes	1	1
≥ 21 dentes	**0,79 (0,67; 0,93)**	**0,79 (0,67; 0,93)**
Autopercepção da saúde bucal		
Muito boa + Boa	1	1
Regular	**0,75 (0,65; 0,88)**	**0,76 (0,65; 0,88)**
Ruim + muito ruim	**0,50 (0,42; 0,61)**	**0,50 (0,42; 0,61)**
Tipo de serviço utilizado		
Privado	1	1
Público	**1,65 (1,05; 2,60)**	2,00 (1,02; 3,91)
Interações entre utilização de serviço público e escolaridade		
Público #0	-	1
Público #1 a 4	-	0,52 (0,26; 1,07)
Público #5 a 8	-	0,65 (0,33; 1,28)
Público #9 a 11	-	0,57 (0,31; 1,08)
Público #≥ 12	**-**	**0,45 (0,22; 0,91)**
Interações entre utilização de serviço público e renda		
Público #>Até 1		
Público #>1 a 3	0,69 (0,0,41; 1,15)	-
Público #>3 a 5	**0,49 (0,29; 0,85)**	-
Público #> 5	**0,50 (0,24; 0,99)**	-
N	29.599	29.599

Estimativas ponderadas pelo delineamento amostral e pelos pesos de amostragem. Estimativas obtidas considerando a amostra dos adultos que usaram os serviços alguma vez na vida. Resultados do modelo do modelo logístico com imputação múltipla (MI). Valores em negrito indicam medidas estatisticamente significativas.
