# Supplementary material for: Use of public and private oral health services by Brazilian adults from 2003 to 2023: trends and socioeconomic inequalities
Source: Rev Bras Epidemiol. 2026 Jul 24;29(Suppl 1):e260006supl1. doi: 10.1590/1980-549720260006.supl.1 (PMC13440361; doi:10.1590/1980-549720260006.supl.1)
Supplement: Translation

# Translation: Utilização de serviços de saúde bucal públicos e privados por adultos no Brasil de 2003 a 2023: tendências e desigualdades socioeconômicas

**DOI:** 10.1590/1980-549720260006.supl.1.2

**Authors:** Líria Sheila Chamane, Rosana Leal do Prado, Doralice Severo Cruz, Ana Luiza Guerra Francisco, Maria Luíza Viana Fonseca, Maria Luiza do Nascimento Silva, Loliza Luiz Figueiredo Houri Chalub, Raquel Conceição Ferreira

**Affiliations:** I Universidade Federal de Minas Gerais - Belo Horizonte (MG), Brasil.; II Ministério da Saúde, Secretaria de Vigilância em Saúde - São Paulo (SP), Brasil.

**Keywords:** Acesso efetivo aos serviços de saúde, Desigualdades de saúde, Serviços de saúde bucal, Fatores socioeconômicos, Equidade, Epidemiologia

## Abstract

**Objetivo:**

Analisar as desigualdades socioeconômicas na utilização de serviços de saúde bucal entre adultos brasileiros de 35-44 anos, no período de 2003 a 2023, segundo escolaridade, renda e tipo de serviço utilizado.

**Métodos:**

Trata-se de um estudo transversal baseado nos dados dos inquéritos nacionais de saúde bucal realizados no Brasil em 2003, 2010 e 2023. Foram estimadas prevalências ponderadas de utilização de serviços de saúde bucal no último ano e calculadas as medidas de desigualdade absoluta (índice angular de desigualdade; IAD) e relativa (índice relativo de desigualdade; IRD), segundo escolaridade e renda. Modelos de regressão logística avaliaram a associação entre indicadores socioeconômicos e utilização de serviços.

**Resultados:**

Entre 2003 e 2023, a proporção de adultos que usaram serviços de saúde bucal no último ano aumentou de 38,7 para 51,6%. O IRD para escolaridade reduziu de 2,16 para 1,39 e o IAD de 0,30 para 0,20, indicando diminuição das desigualdades nesse contexto, estatisticamente significativa entre usuários do serviço público (IRD=1,08; IAD=0,07 em 2023). As desigualdades segundo a renda permaneceram inalteradas. Modelos ajustados mostraram interação significativa entre escolaridade e tipo de serviço, com gradiente mais acentuado no setor privado e mais homogêneo no público.

**Conclusão:**

Entre 2003 e 2023, observou-se aumento na utilização de serviços de saúde bucal no último ano e redução das desigualdades segundo a escolaridade entre usuários dos serviços públicos, sugerindo avanços na consolidação da Política Nacional de Saúde Bucal e na ampliação do acesso público. Entretanto, as desigualdades econômicas persistem, evidenciando a necessidade de políticas redistributivas que integrem equidade e sustentabilidade no financiamento do Sistema Único de Saúde.

## INTRODUÇÃO

A utilização de serviços de saúde bucal é um determinante da saúde, pois reflete o grau em que os recursos disponíveis se traduzem em atenção efetiva^1^ ^,^ ^2^ ^,^ ^3^ ^,^ ^4^. Além de atender às necessidades clínicas, indica a capacidade do sistema de oferecer cuidado oportuno, acessível e contínuo. Quando regular, favorece a prevenção, o diagnóstico precoce e o tratamento das doenças bucais, sendo componente essencial dos sistemas de atenção à saúde. O tipo e a oportunidade de utilização expressam a acessibilidade, a eficiência do sistema e a capacidade dos indivíduos de buscar e obter cuidado^3^ ^,^ ^4^ ^,^ ^5^. Na perspectiva da equidade, constitui indicador de acesso, qualidade e justiça distributiva^5^.

O acesso ao cuidado em saúde bucal é condicionado por fatores sociais, econômicos e estruturais, que vão além das necessidades clínicas^6^ ^,^ ^7^. Persistem gradientes sociais tanto na ocorrência das doenças bucais^7^ ^,^ ^8^ ^,^ ^9^ quanto na utilização dos serviços^9^ ^,^ ^10^. Pessoas com maior escolaridade e renda realizam mais consultas preventivas e reabilitadoras, enquanto grupos socialmente desfavorecidos, apesar das piores condições de saúde bucal, tendem a procurar atendimento apenas em situações de dor ou urgência^7^ ^,^ ^10^ ^,^ ^11^ ^,^ ^12^. Mesmo em contexto de expansão da cobertura pública, como o brasileiro, essas desigualdades persistem^10^, o que reflete o papel da estratificação social e das condições materiais de vida^10^ ^,^ ^13^. Nesse cenário, o sistema de saúde atua como determinante intermediário, com potencial para reduzir iniquidades por meio de ações de promoção e prevenção voltadas aos grupos mais vulnerabilizados^13^.

No Brasil, a atenção à saúde bucal historicamente apresentou forte participação do setor privado^14^ e oferta pública limitada. A criação da Política Nacional de Saúde Bucal (PNSB), em 2004, representou uma inflexão na atuação estatal ao propor um modelo de atenção integral e contínuo no âmbito do Sistema Único de Saúde (SUS)^15^ ^,^ ^16^ ^,^ ^17^ ^,^ ^18^ ^,^ ^19^. Conhecida como Brasil Sorridente e recentemente convertida na Lei 14.572/2023^18^, a política promoveu a ampliação das Equipes de Saúde Bucal na Atenção Primária à Saúde, a criação dos Centros de Especialidades Odontológicas e dos Laboratórios Regionais de Próteses Dentárias, além do fortalecimento de ações de vigilância, como a fluoretação das águas de abastecimento^17^ ^,^ ^18^. Essas iniciativas buscaram garantir atenção integral e reduzir as iniquidades no acesso aos serviços.

Apesar dos avanços na estruturação da atenção pública em saúde bucal nas últimas duas décadas, persistem as desigualdades regionais e socioeconômicas que comprometem a equidade no acesso aos serviços^10^ ^,^ ^11^. A literatura, embora evidencie essas disparidades, ainda carece de estudos que analisem sua evolução temporal com base em dados populacionais representativos^11^ ^,^ ^20^. Análises comparativas entre os inquéritos de saúde bucal permitem avaliar, em perspectiva longitudinal, a possível contribuição das políticas públicas e da expansão da cobertura do SUS para a redução das desigualdades. Este estudo analisou a magnitude e as tendências das desigualdades socioeconômicas na utilização de serviços de saúde bucal entre adultos brasileiros de 35-44 anos, no período de 2003 a 2023, segundo escolaridade, renda e tipo de serviço utilizado, e avaliou se o tipo de serviço modificou a associação entre a utilização do serviço de saúde bucal no último ano de acordo com a escolaridade e a renda.

## MÉTODOS

### Desenho de estudo e fonte de dados

Este é um estudo transversal que analisou dados secundários dos inquéritos nacionais de saúde bucal (SB Brasil), realizados em 2003, 2010 e 2023, no Brasil, reportado segundo a diretriz STROBE (*Strengthening the Reporting of Observational Studies in Epidemiology*). Foram incluídos participantes do grupo etário de 35-44 anos, recomendado pela Organização Mundial da Saúde (OMS) para a avaliação das condições de saúde bucal de adultos^21^. Os três levantamentos utilizaram amostras probabilísticas complexas por conglomerados. Em 2003, os domínios amostrais corresponderam às cinco regiões e aos respectivos municípios, com amostragem em três ou quatro estágios, conforme o porte populacional. As unidades primárias de amostragem (UPA) corresponderam a 50 municípios por região. Em 2010, o plano amostral incluiu 32 domínios (27 capitais e cinco regiões do interior), com amostragem por conglomerados em dois ou três estágios e seleção de 30 UPA por domínio, garantindo coeficiente de variação <15% para estimativas >10%. Em 2023, foram considerados 53 domínios (26 capitais e 27 unidades federativas), adotando-se amostragem em dois estágios (setores censitários e domicílios), com 400 adultos examinados por unidade federativa, assegurando precisão amostral adequada^22^ ^,^ ^23^ ^,^ ^24^ ^,^ ^25^.

### Coleta de dados

Os dados foram coletados nos domicílios, por meio de entrevistas e exames bucais realizados por equipes previamente treinadas, de acordo com os critérios preconizados pela OMS^21^ ^,^ ^23^. Em 2003 e 2010, treinamento e calibração das equipes compreenderam 32 horas de atividades teórico-práticas presenciais. Em 2023, os conteúdos teóricos foram disponibilizados na plataforma Moodle^®^, com uso de vídeos e simulações fotográficas para calibração^26^ ^,^ ^27^. A concordância interexaminadores foi avaliada por coeficiente Kappa ponderado ou simples, com valores mínimos aceitáveis de 0,65 (2003/2010) e 0,61 (2023)^23^ ^,^ ^26^. Os três projetos foram aprovados pela Comissão Nacional de Ética em Pesquisa (2003: 1.356; 2010: 15.498; 2023: 4.823.054). Todos os participantes assinaram o Termo de Consentimento Livre e Esclarecido.

### Variável dependente

O desfecho foi o tempo desde a última consulta ao serviço de saúde bucal. Em 2003 e 2010, o participante respondeu se havia utilizado o serviço de saúde bucal alguma vez na vida. Entre os que utilizaram, o tempo desde a última consulta foi classificado em <1 ano; 1 a 2 anos; e ≥3 anos. Em 2023, uma única pergunta incluiu a opção nunca usou e houve a inclusão da categoria >2 a 3 anos. Para comparabilidade, foi criada uma variável com as categorias: nunca usou; usou no último ano; >1 a 3 anos; e >3 anos.

### Variáveis independentes de interesse e covariáveis

Segundo o modelo de Andersen e Davidson (1997)^3^ ^,^ ^11^ ^,^ ^20^ ^,^ ^28^, as variáveis incluíram fatores facilitadores (renda, escolaridade, tipo de serviço utilizado), predisponentes (sexo, idade e raça/cor da pele) e de necessidade (dor dentária, presença de cárie, perda dentária e autopercepção da saúde bucal). As variáveis independentes de interesse foram renda familiar, escolaridade e tipo de serviço utilizado. A renda familiar foi registrada em reais nos levantamentos de 2003 e 2023 e em categorias pré-definidas em 2010 (até 250; 251-500; 501-1.500; 1.501-2.500; 2.501-4.500; 4.501-9.500; >9.500 reais). Os valores foram convertidos em salários-mínimos (SM), considerando R$ 200,00 (USD 69.56) em 2003, R$ 510,00 (USD 283.41) em 2010 e R$ 1.320,00 (USD 271.71) em 2023, e categorizados em: até 1; >1-3; >3-5; e >5 SM. A variável escolaridade baseou-se nos anos de estudos completos com aproveitamento, sem reprovação, e foi categorizada em 0, 1-4, 5-8, 9-11 e ≥12 anos de estudo^29^ ^,^ ^30^ ^,^ ^31^. O tipo de serviço utilizado, registrado para aqueles que usaram o serviço de saúde bucal alguma vez na vida, foi classificado em serviço público ou privado (privado + convênio ou plano de saúde/privado suplementar e outros).

### Covariáveis

A variável raça/cor da pele foi autorreferida segundo o Instituto Brasileiro de Geografia e Estatística: branca, preta, parda, amarela e indígena. As categorias amarela e indígena foram agrupadas em razão da baixa frequência nos levantamentos.

A dor dentária baseou-se no relato de ocorrência nos últimos seis meses. A presença de cárie dentária foi avaliada segundo os critérios da OMS^21^ e definida como a ocorrência de ≥1 dente com cárie não tratada ou restauração com cárie. A perda dentária baseou-se na contagem de dentes ausentes (perdidos por cárie ou por outras razões) seguindo a OMS^21^, e foi categorizada em <21 e ≥21 dentes presentes, considerando a manutenção de um número de dentes que assegurem as funcionalidades bucais básicas^32^ ^,^ ^33^. A autopercepção da saúde bucal foi padronizada entre os inquéritos. Em 2003, 2010 e 2023, os participantes avaliaram sua saúde bucal ou seu grau de satisfação por meio de escalas equivalentes. Todas as respostas foram reagrupadas em três categorias: positiva (ótima, boa, muito satisfeito, satisfeito), regular (regular, nem satisfeito nem insatisfeito) e negativa (péssima, ruim, insatisfeito, muito insatisfeito).

### Análise estatística

Todas as análises foram realizadas no Stata^®^, considerando o desenho amostral complexo e os pesos amostrais de cada levantamento, incluindo os de 2003 calculados por Queiroz et al.^22^. Foram estimados percentuais de adultos segundo o tempo desde a última consulta ao serviço de saúde bucal, o ano do inquérito e o tipo de serviço (público ou privado) com intervalo de confiança (IC) de 95%. As estimativas por tipo de serviço incluíram apenas adultos que utilizaram serviço de saúde bucal. As respostas “não sei/não respondeu” foram consideradas perdas.

Diferenças absolutas entre anos (Δ2023-2010, Δ2023-2003, Δ2010-2003) foram testadas com o comando lincom^34^. A variação percentual anual (VPA) foi calculada por regressão exponencial do logaritmo dos percentuais em função do tempo (ln(𝑌𝑡)=𝛼+𝛽𝑡, sendo VPA=[exp(𝛽)−1]×100), com IC95% obtidos pelo método delta (nlcom). Os percentuais de adultos que utilizaram serviços no último ano segundo escolaridade e renda foram representados em gráficos *equiplot*. As desigualdades foram mensuradas pelo índice angular de desigualdade (IAD) e pelo índice relativo de desigualdade (IRD)^35^. As categorias ordinais de renda e escolaridade foram convertidas em *ridit-score*, representando a posição socioeconômica cumulativa. Os índices foram estimados por regressão linear generalizada, tendo a utilização de serviços de saúde bucal no último ano como variável dependente e o *ridit-score* como independente, ajustados por todas as covariáveis. Para o IAD, adotou-se a função *identity* e a família binomial; para o IRD, a função log e a família binomial. O IAD expressa a diferença absoluta entre os extremos do gradiente socioeconômico (valores positivos indicam maior prevalência entre grupos favorecidos; e os negativos, entre os desfavorecidos). O IRD representa a razão de prevalência ajustada (IRD>1: maior utilização entre favorecidos; IRD=1: ausência de desigualdade; IRD<1: maior utilização entre desfavorecidos). Ambos foram calculados para amostra total e para subamostras (usuários dos serviços público ou privado). Tendências temporais foram avaliadas por termos de interação entre *ridit-score* e ano do inquérito; coeficientes significativos indicaram mudanças nas desigualdades ao longo do tempo^36^. A associação entre renda, escolaridade e utilização de serviços no último ano foi estimada por regressão logística múltipla, com cálculo de razões de chances e IC95%. Os modelos foram ajustados pelas covariáveis. Um modelo combinado reuniu os três inquéritos, incluindo interações entre ano e variáveis socioeconômicas para testar estabilidade temporal. Como não foram significativas, testaram-se as interações entre tipo de serviço e indicadores socioeconômicos, reunindo os três bancos de dados (modelo 1: tipo de serviço × escolaridade; e modelo 2: tipo de serviço × renda familiar), ajustando-se pelo ano do inquérito. A multicolinearidade foi avaliada pelo fator de inflação da variância (1,03-6,41; média=2,29), sem indicação de colinearidade elevada. O resultado não significativo do teste de Hosmer-Lemeshow indicou bom ajuste, e a área sob a curva ROC demonstrou a capacidade discriminatória global dos modelos^37^.

As probabilidades ajustadas de utilização de serviço no último ano foram estimadas com o comando *margins* e apresentadas graficamente (*marginsplot)*. A análise de sensibilidade comparou modelos com dados completos para todas as variáveis e, após imputação, para variáveis renda e escolaridade. A imputação múltipla por equações encadeadas assumiu *missing at random*, gerou 20 bancos e combinou estimativas e erros-padrão segundo as regras de Rubin^38^ ^,^ ^39^.

## Declaração de disponibilidade de dados:

O conjunto de dados que dá suporte aos resultados deste estudo não está disponível publicamente.

## RESULTADOS

Participaram do estudo 13.430, 9.779 e 9.019 adultos em 2003, 2010 e 2023, respectivamente. As estimativas populacionais, segundo características sociodemográficas e tempo desde a última consulta, para adultos brasileiros, em 2003, 2010 e 2023, estão apresentadas na Tabela 1S (arquivo suplementar). A ausência de resposta para a variável tempo desde a última consulta foi de 0,79%, 1,92% e 3,65%, respectivamente para os anos. Entre os que já haviam utilizado os serviços de saúde bucal (2003: 12.946; 2010: 8.903; e 2023: 8.598), os percentuais de respostas completas para todas as variáveis foram 94%, 93% e 70%, respectivamente. Considerando os três inquéritos (n=31.061), 86,6% apresentaram dados completos (Tabela 2S).

Em todos os inquéritos, a maior parte dos adultos relatou ter utilizado o serviço de saúde bucal no último ano (Tabela 1), porcentagem que aumentou de 38,7% para 51,6% entre 2003 e 2023, enquanto a porcentagem de utilização há >3 anos reduziu de 35,3% para 15,1% (VPA=-3,9%). O crescimento foi significativo entre usuários de serviços públicos, de 37,4% para 52,9% (VPA=+1,74%), enquanto de privados, foi não significativo (VPA=+0,99%) (Tabela 1).

**Tabela 1.** Distribuição percentual de adultos brasileiros segundo o tempo desde a última consulta aos serviços de saúde bucal nos anos de 2003, 2010 e 2023, diferenças dos percentuais entre os anos (Δ) e variação percentual anual para a amostra total e para aqueles que realizaram a última consulta no serviço público ou privado. Brasil.

Tempo desde a última consulta aos serviços de saúde bucal	Inquéritos epidemiológicos			Diferenças entre as estimativas						Variação percentual anual	Valor de p
	2003 (n=13.324)%w (IC95%)	2010 (n=9.591)%w (IC95%)	2023 (n=8.690)%w (IC95%)	Δ2023-2010	Valor de p	Δ2023-2003	Valor de p	Δ2010-2003	Valor de p		
Nunca usou	2,56 (1,95; 3,35)	7,28 (5,74;9,19)	0,89 (0,44; 1,74)	-6,39 (-8,21; -4,57)	<0,001	-1,67 (-2,58; -0,75)	<0,001	4,72 (2,88; 6,57)	<0,001	-6,44% (-20,98; 8,10)	0,385
Último ano	38,69 (37,09; 40,31)	45,80 (43,85; 47,76)	51,62 (48,27; 54,96)	5,83 (1,95; 9,70)	0,003	12,93 (9,22; 16,65)	<0,001	7,11 (4,57; 9,64)	<0,001	+1,39 (0,63; 2,14)	<0,001
>1 a 3 anos	23,44 (22,17; 24,76)	25,76 (23,70; 27,94)	32,35 (29,43; 35,42)	6,59 (2,92; 10,27)	<0,001	8,91 (5,65; 12,18)	<0,001	2,32 (-0,02; 4,81)	0,067	+1,64 (+1,44; 1,85)	<0,001
>3 anos	35,31 (33,58; 37,09)	21,16 (19,50; 22,92)	15,13 (12,91; 17,65)	-6,03 (-8,95; -3,11)	<0,001	-20,18 (-23,13; -17,23)	<0,001	-14,15 (-16,60; -11,70)	<0,001	-3,95 (-6,22; -1,67)	0,001
Tipo de serviço utilizado											
Público											
	2003 (n=6.405)	2010 (n=3.521)	2023 (n=4.130)								
Último ano	37,36 (35,85; 38,88)	42,63 (38,87; 46,38)	52,92 (48,12; 57,72)	10,29 (4,19; 16,39)	0,001	15,55 (10,52; 20,59)	<0,001	5,26 (1,21; 9,31)	0,001	+1,75 (+ 1,63; +1,85)	<0,001
>1 a 3 anos	22,46 (20,74; 24,19)	29,96 (26,58; 33,34)	31,84 (27,87; 35,81)	1,19 (-3,33; 7,10)	0,479	9,38 (5,06; 13,70)	0,001	7,50 (3,70; 11,29)	<0,001	+1,60 (-0,26; 3,44)	0,091
>3 anos	40,17 (38,23; 42,12)	27,41 (24,25; 30,28)	15,24 (11,79; 18,69)	-12,18 (-16,66; -7,69)	<0,001	-24,93 (-28,90; -20,97)	<0,001	-12,76 (-16,23; -9,29)	<0,001	-4,69 (-5,14; -4,24)	<0,001
Privado											
	2003 (n=6.520)	2010 (n=5.358)	2023 (n=4.390)								
Último ano	41,34 (51,26; 56,01)	53,64 (51,26; 56,01)	52,16 (47,68; 56,64)	-1,47 (-6,54; 3,59)	0,568	10,82 (5,69; 15,96)	<0,001	12,30 (8,84; 15,76)	0,001	+0,99 (-0,99; 2,98)	0,327
>1 a 3 anos	25,09 (22,99; 27,19)	26,45 (23,93; 28,96)	33,22 (29,31; 37,13)	6,77 (2,12; 11,43)	0,004	8,13 (3,70; 12,57)	<0,001	1,36 (-1,91; 4,63)	0,415	+1,46 (0,95; 1,97)	<0,001
>3 anos	33,58 (30,98; 36,18)	19,92 (17,85; 21,98)	14,62 (11,38; 17,85)	-5,30 (-9,14; -1,45)	0,007	-18,95 (-23,11; -14,80)	<0,001	-13,66 (-16,98; -10,34)	0,001	-3,86 (-6,30; -1,41)	0,002

Estimativas ponderadas pelo efeito do delineamento amostral e dos pesos de amostragem. As respostas “não sei” e “não respondeu” foram tratadas como perdas, e as porcentagens foram calculadas considerando apenas o total de respostas válidas.

Os gráficos *equiplot* mostraram aproximação dos níveis de escolaridade ao longo do tempo, sugerindo redução das desigualdades na utilização de serviços no último ano (Figura 1, Tabela 3S). Entre usuários do serviço público, a convergência foi mais evidente em razão do aumento da utilização entre os menos escolarizados. O IRD, para a amostra total, reduziu de 2,16 em 2003 para 1,39 em 2023. Entre usuários do serviço público, o IRD diminuiu de 2,17 para 1,08 e o IAD de 0,27 para 0,07, indicando redução das disparidades relativas e absolutas segundo a escolaridade. Entre usuários dos serviços privados, não houve mudanças significativas.

**Figura 1.** *Equiplots* da prevalência de utilização de serviços de saúde bucal no último ano segundo renda familiar e escolaridade para a amostra total e para adultos que realizaram a última consulta nos serviços públicos ou privados. Brasil.

Estimativas ponderadas pelo efeito do delineamento amostral e dos pesos de amostragem.

Em relação à renda, o *equiplot* indicou estabilidade nas distâncias entre estratos socioeconômicos. Para usuários dos serviços privados, observaram-se maiores proporções de utilização de serviços no último ano entre aqueles com maior renda, cujas proporções se distanciaram daqueles com renda de até 1 SM. Para usuários do serviço público, esse padrão também foi notado, mas maiores proporções de utilização no último ano foram observadas no grupo com até 1 SM, comparado àqueles que relataram ter usado o setor privado. Não se observou redução significativa nas desigualdades relativas ou absolutas por renda (Tabela 2, Figura 1).

**Tabela 2.** Índice angular de desigualdade (IAD) e índice de desigualdade relativa (IRD), com intervalos de confiança de 95% (IC95%), para a utilização do serviço de saúde bucal no último ano, segundo escolaridade e renda entre adultos de 35 a 44 anos. Brasil (2003, 2010 e 2023).

População analisada	Inquéritos epidemiológicos			**Coeficiente da interação *ridit-score* × inquérito**	p para tendência
	2003	2010	2023		
ESCOLARIDADE					
	Índice Relativo de Desigualdade (IRD) por escolaridade (IC95%)				
Amostra total	2,16 (1,93; 2,42)	1,56 (1,32; 1,85)	1,39 (1,11; 1,73)	0,80 (0,69; 0,91)	0,001
Utilizou serviço público	2,17 (1,92; 2,46)	1,27 (0,97;1,65)	1,08 (0,83; 1,40)	0,74 (0,64; 0,85)	<0,001
Utilizou serviço privado	2,19 (1,91; 2,51)	1,58 (1,25; 2,02)	1,73 (1,20; 2,48)	0,89 (0,70; 1,13)	0,339
	Índice Angular de Desigualdade (IAD) por escolaridade (IC95%)				
Amostra total	0,30 (0,25; 0,34)	0,22 (0,14; 0,30)	0,20 (0,08; 0,31)	-0,06 (-0,13; 0,004)	0,067
Utilizou serviço público	0,27 (0,22; 0,33)	0,11 (0,004; 0,23)	0,07 (-0,08; 0,22)	-0,08 (-0,16; -0,02)	0,019
Utilizou serviço privado	0,28 (0,22; 0,34)	0,25 (0,13; 0,36)	0,26 (0,08; 0,45)	-0,02 (-0,13; 0,08)	0,663
RENDA					
	Índice Relativo de Desigualdade (IRD) por renda (IC95%)				
Amostra total	1,82 (1,54; 2,15)	1,63 (1,37; 1,93)	1,61 (1,31; 1,98)	0,91 (0,79; 1,04)	0,176
Utilizou serviço público	1,34 (1,17; 1,61)	1,11 (0,85; 1,43)	1,24 (0,89; 1,72)	0,95 (0,79; 1,14)	0,566
Utilizou serviço privado	2,09 (1,60; 2,74)	1,72 (1,43; 2,07)	2,15 (1,53; 3,02)	0,97 (0,76; 1,23)	0,798
	Índice Angular de Desigualdade (IAD) por renda (IC95%)				
Amostra total	0,24 (0,18; 0,30)	0,25 (0,17; 0,33)	0,25 (0,14; 0,37)	-0,01 (-0,07; 0,06)	0,991
Utilizou serviço público	0,12 (0,05; 0,18)	0,05 (-0,07; 0,16)	0,15 (-0,06; 0,36)	0,04 (-0,10; 0,11)	0,944
Utilizou serviço privado	0,30 (0,20; 0,39)	0,28 (0,19; 0,37)	0,38 (0,23; 0,55)	0,02 (-0,09; 0,12)	0,728

Estimativas ponderadas pelo delineamento amostral e pesos de amostragem. Modelos ajustados por sexo, idade, raça/cor da pele, dor dentária no último mês, cárie dentária, número de dentes presentes e autopercepção de saúde bucal. Respostas “não sabe” e “não respondeu” foram tratadas como perdas, e as proporções foram calculadas considerando apenas respostas válidas.

A regressão logística bruta mostrou associação significativa entre renda, escolaridade e utilização de serviços (Tabela 4S). Não houve interação significativa entre ano e indicadores socioeconômicos, indicando estabilidade temporal dos efeitos (Tabela 5S).

No modelo ajustado, observaram-se interações significativas entre tipo de serviço e ambos os indicadores, evidenciando que o efeito de renda e escolaridade variou conforme o tipo de serviço utilizado. No modelo 1 (escolaridade × tipo de serviço), a probabilidade de utilização no último ano aumentou com a escolaridade tanto entre usuários de serviços públicos como privados, sendo o gradiente mais acentuado entre usuários de serviços privados, variando de 21,35% (0 anos de estudo) a 55,06% (≥12 anos). Entre usuários de serviços públicos, variou de 34,75% a 52,41%, com linha mais horizontal, indicando menor desigualdade. No modelo 2 (renda×tipo de serviço), a probabilidade de utilização no último ano entre usuários do serviço público variou de 46,39% (≤1 SM) a 54,77% (>5 SM), enquanto do serviço privado variou de 33,97% a 58,95%. Mesmo com baixa renda, usuários do serviço público apresentaram maior probabilidade de utilização no último ano em relação aos de renda semelhante que usaram o serviço privado (Tabela 3, Figura 2).

**Tabela 3.** Associação entre escolaridade ou renda e a utilização de serviços de saúde bucal no último ano entre adultos de 35 a 44 anos. Brasil (2003, 2010 e 2023).

Variáveis	Utilização de serviços de saúde bucal no último ano	
	Modelo 1 (inclusão da interação entre utilização de serviço público × escolaridade)	Modelo 2 (inclusão da interação entre utilização de serviço público × renda familiar)
	OR (IC95%)	OR (IC95%)
*Ano do levantamento*		
2003	1	1
2010	1,46 (1,28; 1,65)	1,46 (1,29; 1,65)
2023	1,68 (1,30; 2,17)	1,68 (1,30; 2,17)
*Idade*	0,98 (0,95; 1,00)	0,98 (0,96; 1,00)
*Sexo*		
Masculino	1	1
Feminino	1,39 (1,20; 1,61)	1,41 (1,22; 1,63)
*Raça/Cor da pele*		
Branca	1	1
Preta	0,99 (0,83; 1,20)	0,99 (0,82; 1,19)
Amarela + indígena	0,99 (0,75; 1,32)	0,99 (0,74; 1,32)
Parda	0,95 (0,82; 1,10)	0,94 (0,82;1,09)
*Renda familiar (salários-mínimos)*		
Até 1	1	1
>1 a 3	1,21 (1,01; 1,47)	1,58 (1,04; 2,38)
>3 a 5	1,64 (1,34; 2,00)	2,39 (1,65; 3,44)
>5	2,20 (1,71; 2,83)	3,06 (2,06; 4,53)
*Escolaridade (anos de estudo)*		
0	1	1
1 a 4	2,58 (1,39; 4,78)	1,66 (1,23; 2,24)
5 a 8	2,62 (1,49; 4,62)	1,90 (1,43; 2,52)
9 a 11	4,01 (2,28; 7,05)	2,74 (1,99; 3,79)
≥12	4,97 (2,67; 9,29)	3,10 (2,21; 4,35)
*Dor dentária nos últimos 6 meses*		
Não	1	1
Sim	2,28 (1,96; 2,64)	2,26 (1,95; 2,62)
*Presença de cárie dentária*		
Ausente	1	1
≥1 dente cariado	0,84 (0,74; 0,96)	0,84 (0,74; 0,96)
*Número de dentes presentes*		
<21 dentes	1	1
≥21 dentes	0,76 (0,64; 0,92)	0,76 (0,64; 0,92)
*Autopercepção da saúde bucal*		
Positiva	1	1
Regular	0,81 (0,69; 0,95)	0,80 (0,69; 0,94)
Negativa	0,52 (0,43; 0,63)	0,52 (0,42; 0,63)
*Tipo de serviço utilizado*		
Privado	1	1
Público	2,04 (1,07; 3,90)	1,76 (1,09; 2,84)
*Interações entre utilização de serviço público e escolaridade (anos)*		
0	1	
1 a 4	0,52 (0,26; 1,04)	-
5 a 8	0,65 (0,33; 1,26)	-
9 a 11	0,58 (0,32; 1,05)	-
≥12	0,44 (0,21; 0,90)	-
*Interações entre utilização de serviço público e renda (salários-mínimos)*		
Até 1		
>1 a 3	-	0,66 (0,39; 1,13)
>3 a 5	-	0,45 (0,26; 0,77)
>5	-	0,47 (0,23; 0,95)
*N*	28.045	26.902

IC: intervalo de confiança; OR: *odds ratio* (razão de chances).

Estimativas ponderadas pelo delineamento amostral e pelos pesos de amostragem. Estimativas obtidas considerando a amostra dos adultos que utilizaram os serviços de saúde bucal alguma vez na vida. Valores em negrito indicam medidas estatisticamente significativas.

**Figura 2.** Probabilidades preditas ajustadas de utilização de serviços de saúde bucal no último ano segundo escolaridade e renda, de acordo com o tipo de serviço utilizado.

Probabilidades (Pr) preditas a partir do modelo de regressão logística ajustado. Modelo 1: (inclusão da interação entre utilização de serviço público×escolaridade), ajustado pelas variáveis ano do inquérito, sexo, idade, renda, cor da pele, dor dentária, autopercepção da saúde bucal e número de dentes presentes. Modelo 2 (inclusão da interação entre utilização de serviço público × renda familiar), ajustado pelas variáveis ano do inquérito, sexo, idade, escolaridade, cor da pele, dor dentária, autopercepção da saúde bucal e número de dentes presentes. Estimativas ponderadas pelo delineamento amostral e pelos pesos de amostragem. Estimativas obtidas considerando a amostra dos adultos que utilizaram os serviços de saúde bucal alguma vez na vida.

O desempenho dos modelos foi satisfatório, com bom ajuste global (teste Hosmer-Lemeshow modelo 1: F(9,1782)=0,67; p=0,7399; modelo 2: F(9,1782)=1,17, p=0,3099). A AUC ROC foi de 0,6661 (0,65981; 0,67247) para o modelo 1 e de 0,6580 (0,65155; 0,66450) para o modelo 2, evidenciando capacidade discriminatória moderada. Os coeficientes dos modelos com dados imputados foram semelhantes aos obtidos com a amostra de casos completos para todas as variáveis (Tabela 6S).

## DISCUSSÃO

Embora persistam gradientes sociais no acesso aos cuidados odontológicos, este estudo evidenciou aumento na utilização dos serviços de saúde bucal no último ano no Brasil entre 2003 e 2023 e redução das desigualdades nessa utilização, segundo a escolaridade. A análise estratificada por tipo de serviço evidenciou redução dessas desigualdades entre usuários dos serviços públicos. Já no setor privado, o gradiente educacional permaneceu mais acentuado, refletindo padrões de estratificação social. Esses achados sugerem efeito positivo da consolidação da PNSB no âmbito do SUS e sua contribuição para a ampliação do acesso, especialmente por meio da expansão das Equipes de Saúde Bucal na Atenção Primária à Saúde e da estruturação da Rede de Saúde Bucal^16^ ^,^ ^17^ ^,^ ^40^.

Por outro lado, os resultados revelaram menor utilização dos serviços no último ano entre indivíduos com maiores demandas clínicas, como presença de cárie e autopercepção negativa da saúde bucal, sugerindo acesso tardio, acúmulo de necessidades e persistência de barreiras entre grupos mais vulneráveis. Esse padrão é corroborado pela maior utilização dos serviços entre pessoas que relataram dor dentária. A maioria dos participantes buscou atendimento por dor, necessidade de extração ou de tratamento. Contudo, entre usuários do serviço público em 2023, cerca de 33% relataram consulta de rotina como motivo principal, um avanço expressivo em relação aos 19% observados em 2010 (resultados não apresentados). Esse achado evidencia progressos na incorporação de ações preventivas no SUS, ainda que o padrão curativo permaneça predominante.

Nesse contexto, a expansão da cobertura e o planejamento das políticas públicas devem considerar a lei do cuidado inverso, garantindo que o acesso e a qualidade do cuidado não privilegiem os estratos mais favorecidos, mas priorizem grupos com maiores necessidades^41^ ^,^ ^42^. O SUS desempenha papel central ao se contrapor à lógica de mercado, que tende a reforçar desigualdades e perpetuar os efeitos dessa lei. O aumento da busca por serviços voltados à manutenção da saúde bucal pode também refletir mudanças culturais no comportamento em saúde, uma vez que indivíduos que realizam consultas periódicas tendem a adotar práticas de autocuidado mais consistentes, reduzindo fatores de risco e ampliando o capital de saúde bucal ao longo da vida^7^ ^,^ ^8^. A tendência de aumento na utilização dos serviços de saúde bucal no último ano e a busca pelo cuidado em tempo oportuno podem contribuir para modificar o perfil epidemiológico da população, favorecendo práticas assistenciais mais conservadoras e voltadas à promoção da saúde e à prevenção de agravos. Além disso, esse processo pode reduzir a necessidade de procedimentos invasivos e fortalecer a transição para um modelo de atenção à saúde com perspectiva mais integral^30^.

A análise revelou diferenças consistentes na utilização de serviços de saúde bucal no último ano, segundo escolaridade e renda, com aumento da prevalência entre 2003 e 2023. A redução das desigualdades segundo a escolaridade, sobretudo entre usuários do serviço público, indica o papel redistributivo do SUS e o avanço das políticas universais de saúde bucal. Esse resultado sugere superação parcial de barreiras educacionais, impulsionada pela expansão da Atenção Primária à Saúde e pela interiorização dos serviços especializados^20^ ^,^ ^30^. Esse processo foi fortalecido pela recomposição do financiamento federal da PNSB em 2023, via Portaria GM/MS 1.924/2023^43^, que reajustou incentivos às Equipes de Saúde Bucal, Laboratórios Regionais de Próteses Dentárias e Centros de Especialidades Odontológicas, contribuindo para reverter o subfinanciamento histórico, ampliar a capacidade de resposta do SUS, especialmente em municípios menores e regiões mais vulneráveis, e sustentar ganhos de equidade e resolutividade^44^.

Esses achados convergem com evidências de que, ao reorganizar o modelo de atenção em saúde bucal, os sistemas universais conseguem diminuir as iniquidades ligadas à educação, ainda que persistam disparidades econômicas^28^. As desigualdades por renda permaneceram praticamente inalteradas nas duas décadas analisadas. Apesar da ampliação da oferta pública, a renda familiar continuou a influenciar a utilização dos serviços, sobretudo os privados, confirmando o papel dos fatores capacitadores, segundo os quais a capacidade econômica é determinante essencial do acesso. Barreiras financeiras diretas e indiretas, como custos de transporte, perda de dias de trabalho e gastos materiais, ainda limitam a utilização de serviços pelos grupos de menor renda, restringindo o potencial das políticas públicas de promover equidade econômica^3^.

Os efeitos da renda sobre a redução das iniquidades são complexos e refletem as limitações dos mecanismos de redistribuição no contexto brasileiro. Embora programas como o Bolsa Família tenham contribuído para reduzir a pobreza e melhorar os indicadores de saúde e educação^45^, a concentração de renda no país permanece entre as maiores do mundo. Parte dessa persistência decorre da estrutura tributária regressiva, que onera desproporcionalmente os mais pobres, e do aumento da informalidade, que reduz a arrecadação e enfraquece a proteção social^46^ ^,^ ^47^. Nesse cenário, a renda segue como um determinante estrutural das iniquidades em saúde^13^, influenciando de forma desigual o acesso aos serviços de saúde bucal e a utilização efetiva do cuidado. Políticas de justiça fiscal e ampliação do investimento social - saúde, educação e proteção social - desempenham papel fundamental na mitigação das desigualdades^46^.

As limitações incluem possível viés de memória decorrente de informações autorreferidas e ausência de variáveis contextuais no modelo. A amostragem por conglomerados pode subestimar erros-padrão em subgrupos pequenos, além de possibilitar sub-representação de indivíduos sem acesso aos serviços. As análises baseiam-se em três momentos, o que reduz o poder para detectar mudanças graduais nas desigualdades. Os testes de interação entre *ridit-score* e ano avaliam variações estatísticas ao longo do tempo, mas não possibilitam inferências causais nem a identificação de mecanismos subjacentes, como o impacto específico de políticas públicas ou outras mudanças contextuais. O cálculo da VPA pressupõe padrão exponencial constante e pode não capturar descontinuidades decorrentes de alterações econômicas ou institucionais. As desigualdades foram mensuradas pelo IRD e IAD, dada sua comparabilidade temporal ao considerar a posição relativa das categorias socioeconômicas^35^ ^,^ ^36^ ^,^ ^48^. Embora mudanças demográficas possam afetar a composição populacional, esses índices são adequados para análises de tendência por incorporarem toda a distribuição socioeconômica. Utilizou-se a renda familiar por ser compatível entre os inquéritos. Reconhece-se, contudo, que a renda *per capita* equivalente é recomendada para estratificação econômica e análise de pobreza, com impactos nas associações renda-saúde bucal^49^. Contudo, sua aplicação foi inviabilizada pelas diferentes formas de coleta da renda (2010: categórica; 2003/2023: contínua), comprometendo a comparabilidade temporal, objetivo central da análise.

Entre 2003 e 2023, observou-se aumento na utilização de serviços de saúde bucal no último ano e redução das desigualdades segundo escolaridade, sugerindo avanços na consolidação da PNSB e na ampliação do acesso público. Entretanto, as desigualdades econômicas persistem, evidenciando a necessidade de políticas redistributivas que integrem equidade e sustentabilidade no financiamento do SUS.

## Material suplementar
